# Investigating the exhaled lipid biomarkers among e-cigarette users compared to non-smokers

**DOI:** 10.1186/s12944-026-02911-8

**Published:** 2026-02-27

**Authors:** Shanzina Iasmin Sompa, Per Larsson, Jie Ji, Bengt Sjögren, Swapna Upadhyay, Koustav Ganguly, Mats Josefson, Anna Bergström, Anna-Carin Olin, Lena Palmberg

**Affiliations:** 1 Institute of Environmental Medicine, Karolinska Institutet, Stockholm, 17177 Sweden; 2 School of Public Health and Community Medicine, Gothenburg University, Gothenburg, Sweden; 3 Chalmers Mass Spectrometry Infrastructure, Department of Life Sciences, Chalmers University of Technology, Gothenburg, Sweden; 4 Global Product Development, Pharmaceutical Technology and Development, Operations, AstraZeneca, Gothenburg, Sweden; 5 Institute for Integrative and Innovative Research (I³R), University of Arkansas, Fayetteville, AR USA; 6 Integrative Toxicology, Institute of Environmental Medicine, Karolinska Institutet, Stockholm, 17177 Sweden

**Keywords:** e-cigarettes, PExA, Lipid class, Lipid species, IL-13

## Abstract

The Particle in Exhaled Air (PExA) method provides a non-invasive way to evaluate how electronic (e)-cigarette aerosols affect the small airways by providing a sample of their lining fluid. This study explored for the first time the changes in the lipid profiles of airway lining fluid and their links to systemic inflammatory marker IL-13-producing T cells in blood, local airway type Ⅱ inflammatory marker FeNO, and local innate immune marker TLR2 in sputum among e-cigarette users, cigarette smokers, and non-smokers.

PEx samples were collected on a single occasion from 24 non-smokers, 21 cigarette smokers, and 17 e-cigarette users aged 20 to 65 years. Participants completed a questionnaire including information on cigarette and e-cigarette use. All participants had normal lung function (FEV_1_/VC ≥ 0.7) and no history of allergy or lung diseases. Statistical significances were tested by the Kruskal-Wallis test followed by the Mann-Whitney test as post-hoc, linear regression, and OPLS-DA analysis.

A total of 86 lipid species across 9 lipid classes were identified in the analysis. Significant differences in both phospholipid classes and individual lipid species were found among e-cigarette users, cigarette smokers, and non-smokers, with the most pronounced differences between e-cigarette users and non-smokers. Notably, the percentages of lysophosphatidylcholine (LPC), sphingomyelin (SM), and phosphatidylethanolamine (PE) lipid classes were higher in e-cigarette users compared to non-smokers. Additionally, a strong association was identified between IL-13-producing T cells and lipid profiles in e-cigarette users compared to non-smokers.

The observed changes in lipid profiles among e-cigarette users may indicate disruptions in lipid homeostasis linked to chronic inflammatory lung diseases. This underscores the need for further research on the long-term effects of e-cigarette use, as the precise implications remain unclear.

**Supplementary Information:**

The online version contains supplementary material available at 10.1186/s12944-026-02911-8.

## Introduction

Since 2003, electronic (e)-cigarettes have gained popularity as an alternative to traditional cigarettes. This shift is largely attributed to the perception that they offer reduced harm and their potential role in smoking cessation [1]. While traditional cigarettes are well-known as a significant contributor to lung diseases, the long-term health effects of e-cigarettes are still being researched. Growing evidence suggests that e-cigarette use may have considerable physiological and health implications, especially for respiratory health [1–6]. For example, a recent meta-analysis showed an increased risk of developing COPD in e-cigarette users compared with non-users [7]. In addition, higher levels of fractional exhaled nitric oxide (FeNO), interleukin-13 (IL-13), and surface toll-like receptor 2 (TLR2) expressions, essential indicators of local airway and systemic inflammation, were reported among e-cigarette users compared to non-smokers [6]. At the cellular levels, e-cigarettes are also known to be associated with cytotoxicity, oxidative stress, inflammation, and lipid alteration [5, 8]. Assessing exhaled biomarkers can provide valuable insights into airway inflammation, oxidative stress, and overall lung health [9].

The Particle in Exhaled Air (PExA) technique is an innovative and non-invasive method for collecting small airway samples, specifically targeting lipid biomarkers from the lower respiratory tract [10]. Unlike exhaled breath condensate (EBC), which collects condensate from the entire airway [11], PExA is mechanistically distinct. These non-volatile particles primarily originate from the airway lining fluid (ALF) of small airways, which is crucial for maintaining lung homeostasis and safeguarding against inhaled pollutants [12]. ALF from small airways is rich in lipid constituents such as phospholipids and sphingolipids, of which phospholipids are key components of pulmonary surfactant. The most abundant phospholipid is phosphatidylcholine (PC), which is usually present in desaturated form, such as di-palmitoyl-phosphatidyl-choline (DPPC). Phosphatidylglycerol (PG) is the second most abundant phospholipid in the surfactant. Other minor phospholipids in the surfactant include phosphatidylethanolamine (PE), sphingomyelin (SM), phosphatidylinositol (PI), and phosphatidylserine (PS).

Sphingolipids, such as ceramide, glucosylceramide (GlcCer), sphingosine, and sphingosine-1-phosphate (S1P), are essential for the structural organization of cell membranes. They also act as potent bioactive mediators that regulate apoptosis, inflammation, and the functionality of epithelial barriers, and GlcCer, in particular, is associated with conditions like emphysema and COPD [13, 14]. Additionally, ALF contains cholesterol and other lipid mediators that contribute to immune responses and inflammatory regulation [15–18]. Alterations in the surfactant lipids are associated with the development of lung diseases [15, 19, 20], and this is further impacted by cigarette smoking, which is linked to changes in both surfactant lipids and proteins in the lungs [21].

PExA provides the opportunity for a better understanding of the effects of inhaled substances, such as e-cigarette aerosols, which directly interact with the epithelial surface of the respiratory tract. In addition, PEx (exhaled particles) samples have never been used to study the alterations in the lining fluid among e-cigarette users. Therefore, this study aims to explore the composition of surfactant phospholipids using PEx samples among e-cigarette users and cigarette smokers compared to non-smokers. Moreover, we wanted to assess if the surfactant phospholipids are influenced by the changes in local airway type Ⅱ inflammatory marker FeNO, local innate immune marker TLR2 in sputum, and systemic inflammatory marker IL-13-producing T-cells in blood in these groups.

## Methods

### Study design and study participants

All participants were recruited through advertisement, and the study was conducted between October 2019 and April 2024. Due to COVID-19, the study was postponed from April 2020 to October 2021. From October 2021 onwards, vaccination against COVID-19 was demanded for participation. The eligibility criteria and recruitment procedures were unchanged, ensuring balanced groups, except for vaccination status due to public health considerations. In addition, the sample sizes for both pre- and post-pandemic recruitment are roughly balanced across the three groups.

A flowchart illustrating the participant recruitment process is shown in Figure S1. All samples were collected on a single occasion per person. PEx samples were collected from 24 non-smokers, 21 cigarette smokers, and 17 e-cigarette users. For the analysis of IL-13-producing T cells in blood and FeNO, we included data from 22 non-smokers, 20 cigarette smokers, and 20 e-cigarette users. For the analysis of TLR2 in immune cells from induced sputum, we only included samples from 16 non-smokers, 15 cigarette smokers, and 15 e-cigarette users that contained less than 30% squamous cells.

The current study was part of a clinical study that used a self-reported questionnaire to ascertain a minimum daily use of e-cigarettes for at least 1 year. More detailed information on the eligibility criteria and other health parameters assessed in the participants has previously been described [6]. In short, all participants were aged between 20 and 65 years, had normal lung function with a forced expiratory volume in one second (FEV_1_)/vital capacity (VC) value ≥ 0.7 at baseline, and no history or sign of allergy and/or lung and airway diseases.

### Sample collection and analysis

#### Collection of PEx samples

PEx was collected using the PExA Instrument (PExA AB, Gothenburg, Sweden). Participants breathed through a mouthpiece with a two-way (non-rebreathing) valve, allowing particle-free air to be inhaled and exhalations to be diverted back to the room (in between breathing manoeuvres) or to the PExA instrument (thermostated to 36 °C to eliminate condensation). A nose clip was used during the procedure so that only particle-free air from the mouthpiece was inhaled. A standardized breathing manoeuvre that induces airway closure and re-opening in small airways was used when collecting particles from breath. Detailed procedures for performing deep breathing manoeuvres and PEx (exhaled particles) sample collection have been described previously [22, 23].

The PExA instrument collects particles from the exhaled aerosol using the cascade impactor method. This method is based on the inertia of particles and allows particles of a selected size interval to be collected. A hydrophilic polytetrafluoroethylene (PTFE) membrane (0.45 μm pore size, 25 mm diameter, Merck Millipore Ltd., Cork, Ireland) was used as the sampling substrate inside the impactor. The PexA instrument contains an optical particle counter (Grimm Aerosol Technik GmbH & Co, Ainring, Germany) to determine the collected particle amount online during the collection.

Before sample collection, participants were asked to inhale HEPA-filtered air for 2 min. The sampling session started with a full exhalation to residual volume, breath holding for 5 s, then a rapid inhalation to total lung capacity, followed by normal exhalation. Only the last exhalation was sampled by opening the second valve. The procedure was performed for a maximum of 15 min or until 120 ng particles were collected (calculated from the optical particle counter data), and the membranes were stored in cryovials at -80 ͦ C until analysis, which is a standard practice in lipidomics, effectively preserving major lipid classes over prolonged durations [24, 25]. All PEx samples were analyzed concurrently to minimize batch effects. The number of particles exhaled in the size range of 0.5–5 μm was registered during the exhalation and presented as particle mass/L. The instrument is equipped with a non-rebreathing valve to prevent participants from inhaling air from the device. In the rare case of a valve malfunction, any residual air is purged before the next collection. Droplets from participants stick to the impactor surfaces permanently, with sample sizes being very small (~ 100 ng per collection, equating to about 10 million samples per gram). Blank measurements confirm no carry-over of aerosolized particles or lung lipids. The air nozzle areas of the impactor are regularly cleaned, and the instrument is flushed with dry air between collections to prevent microbial growth. Participants were asked to abstain from food and drink for at least two hours before their laboratory visit to minimize the effects of ingestion on exhaled particle generation. PExA sampling took place approximately two hours after arrival, following all other examinations (see Figure S1). This timing and pre-sampling restriction aimed to reduce variability in particle number and mass, while recognizing that factors like breathing patterns, airway dynamics, and behavioural habits (such as smoking or vaping techniques) may still influence exhaled particle output.

### Fractional nitric oxide in exhaled air (FeNO)

A detailed procedure has previously been described [6]. In brief, FeNO was measured by the NIOX VERO (Circassia AB, Uppsala, Sweden) device following the manufacturer’s instructions and the ATS/ERS guideline [26]. Two consecutive measurements were taken, and the mean value was used for analysis.

### Collection of blood and sputum samples

The procedures for collecting blood and induced sputum samples, as well as analysis of IL13-producing T cells in blood and surface expression of TLR2 in sputum immune cells using flow cytometry, have previously been described [6, 27]. In brief, peripheral blood was collected in vacutainer tubes (BD Vacutainer, Swemed). Sputum was induced by inhalation of saline at increasing concentrations (0.9%, 3%, 4%, 5%) using an ultrasonic nebulizer (De Vibliss Ultraneb 2000) following inhalation of salbutamol (0.4 mg). The sample was considered adequate when it was more than 2 g and macroscopically appeared to be saliva-free. After processing with 0.1% dithiothreitol, total cell counts and viability were performed with trypan blue staining. Sputum samples containing less than 30% squamous cells were considered successful and included in the analysis.

The flow cytometric acquisition was performed by LSR Fortessa™ (BD Bioscience, US). The data were analysed using FlowJo software (BD Biosciences, US).

### Lipid analysis

A detailed procedure for analysing surfactant lipids from PEx samples of small airways has been described previously [28]. In brief, isotope labelled internal standards from Avanti Research (SPLASH lipid mix SKU:330707 and GlucosylCeramide SKU:860638) were spiked to each sample membrane and allowed to dry. The sampling membrane was placed in centrifugal filter inserts (Millipore UFC30LG25), and lipids were extracted using organic extraction solvents inside a thermomixer at a temperature of 35 °C and shaking at 300 rpm. The eluate was recovered to the bottom of the microtube by centrifugation, while the sampling membrane remained in the filter insert. The procedure was first done with isopropanol and then repeated with methanol in the same filter insert. The methanol and isopropanol eluates in the microtube were transferred into LC vials (Waters Total Recovery vial SKU: 186002805) and stored at minus 20 °C before analysis. Shortly before analysis, the solvent in the LC-vials was evaporated under a stream of nitrogen, and the remaining lipid film was solubilised in 100 µl injection solvent consisting of acetonitrile and isopropanol (ratio 2:1). The extracted lipids were analysed with a targeted LC-MSMS method using the Waters ACQUITY UPLC I-Class chromatography system coupled to an electrospray ionization source on a Waters Xevo TQ-XS triple quadrupole mass spectrometer detector (Milford, MA, USA). The mass detector was using the Multiple Reaction Monitoring (MRM) mode for data acquisition. PC/PG/PI/PE lipids were analysed in negative mode monitoring both fatty acyl fragments, one was selected as a quantifier ion and the other as a confirmation ion.

Quantification was based on the peak area of the analyte divided by the peak area of the internal standard, the response factor. The response factor was multiplied with the mol of internal standard spiked to the sample. One internal standard per lipid class was used. The results of the lipid analyses were calculated as relative amount i.e., mol%, where the sum of all species will be 100%, both at the species level (mol%=(species_mol)/(lipid_sum_mol)) and at the leach lipid class level (mol%=(class_sum_mol)/(lipid_sum_mol)). Type II isotope correction factors were calculated with the online correction tool LICAR, which is described by Gao et al. [29]. When isomers were found as two distinct peaks, then the first peak was annotated with and _A and the second with a _B. For example, LPC(16:1)_A and LPC(16:1)_B correspond to the SN1 and SN2 positions of the 16:1 fatty acyl group on the glycerol backbone.

To ensure the reliability and reproducibility of lipidomic measurements from PExA samples, several validation steps were performed. Linearity between PEx Mass and Lipid amount was evaluated by showing that sampling of increasing PEx mass resulted in proportional increases in lipids. Precision was assessed by splitting sample membranes in half before extraction and analysis. Analyte identity was verified using confirmation ions and isotopic patterns. Extraction efficiency was optimized using different solvents. We also included repeated HQC LQC and Zero samples in each run to verify each batch for both extraction and analysis, as well as to monitor batch-to-batch consistency. In this batch, median RSD% for the HQC (*n* = 3) sample and mol% variable was 6.66% for both extraction and analysis.

### Statistics

All data were analysed, and results were generated using STATA 16 (Statacorp, TX, USA) and SIMCA 17.0.2 software (SIMCA, Sartorius AG, Germany). Using Simca, the proportion of lipid classes and lipid species was analysed using unit variance (UV)-scaling of the variables.

The results are presented as medians with interquartile range, means with standard deviation, means with 95% confidence interval, or regression coefficients with 95% confidence interval. The median comparison between groups was assessed using a non-parametric Kruskal-Wallis signed-rank test, followed by the Mann-Whitney test as a post-hoc test. Linear regression analysis was performed to identify the differences in the proportion of lipid classes and individual lipid species in e-cigarette users and cigarette smokers, using non-smokers as a reference group. In addition, linear regression models with interaction terms were used to determine whether the association between phospholipid classes and FeNO, IL-13-producing T cells in blood, and surface TLR2 expression in sputum immune cells differed among e-cigarette users and cigarette smokers compared to non-smokers. A *P* value of < 0.05 was considered significant. Significant pairwise differences following Kruskal-Wallis tests were adjusted for multiple comparisons using Dunn’s post hoc test, and significant differences were defined as adjusted *P* values < 0.05. Significant *P* values from linear regression analysis were adjusted for False Discovery Rate (FDR) using the Benjamini-Hochberg (BH) procedure, and Q values (adjusted *P* values) < 0.05 were considered significant.

Multivariate analysis was conducted using principal component analysis (PCA) and orthogonal projections to latent structure discriminant analysis (OPLS-DA) [30] to evaluate the differences in the composite profile of lipid classes and individual lipid species between non-smokers, cigarette smokers, and e-cigarette users. OPLS regression was used to identify the differences in the profile of lipid classes among e-cigarette users, cigarette smokers, and non-smokers. OPLS-DA was also used to detect the differences in the profile of phospholipid species with FeNO, IL-13-producing T cells in blood, and surface TLR2 expression in sputum immune cells in the investigated groups.

## Results

### Demographic characteristics of the study population

Demographic characteristics of the study participants are presented in Table 1. The characteristics of the participants in the study were generally similar between non-smokers, cigarette smokers, and e-cigarette users. However, the proportion of females differed across the groups, with 66.6% among cigarette smokers, 52.9% among e-cigarette users, and 41.6% among non-smokers, although not statistically significant (*P* = 0.2), reflecting differences among all three groups collectively. The median total number of exhaled particles varied significantly among cigarette smokers (113 ng/L), e-cigarette users (63 ng/L), and non-smokers (83 ng/L), with a *P* value of 0.03 indicating a statistically significant overall difference. However, pairwise comparisons showed no significance when comparing individual groups to non-smokers (results not shown). Participants reported a median e-cigarette usage of 2.7 years (IQR 2–3.2), whereas cigarette smokers indicated a median pack-year history of 5 years (IQR 2.7–12).

**Table 1:** Demographic characteristics of the study population

	Non-smokers *N* = 24	Cigarette smokers *N* = 21	E-cigarette users *N* = 17	*P* value
Age, years, median (IQR)	27 (24, 36)	30 (21, 39)	26 (16, 29)	0.3
Female^$^, n (%)	10 (41.6)	14 (66.6)	9 (52.9)	0.2
BMI, kg/m2, median (IQR)	23.5 (21.6, 26.3)	26.3 (21.4, 27.8)	23 (20, 26.2)	0.8
% pred FEV_1_^#^, mean (SD)	98 (10.7)	97 (10.9)	99 (12)	0.8
% pred VC^#^, mean (SD)	92 (9.3)	91 (12.5)	91.4 (12.7)	0.9
FEV_1_/VC^#^, %, mean (SD)	87 (5.7)	87.2 (8.9)	90.6 (7.1)	0.2
Total accumulated particle in exhaled air, ng/L, median (IQR)	83 (37, 123)	113 (68, 126)	63 (31, 88)	**0.03**
E-cigarette use, total year, median (IQR)	-	-	2.7 (2, 3.2)	
Cigarette smoking, pack years, median (IQR)	-	5 (2.7, 12)	-	
	*N* = 22	*N* = 20	*N* = 20	
FeNO^§^, ppb, median (IQR)	11.5 (7.5, 16.7)	9 (6.5, 11)	14.5 (11.0, 23.0)	**0.006**
IL13^§^ producing CD3 + T cells in blood, %, median (IQR)	2.6 (1.1, 5.2)	6.6 (1.9, 54.6)	62.7 (25.8, 75.0)	**< 0.001**
	*N* = 16	*N* = 15	*N* = 15	
TLR2^§^ expression on sputum immune cells, MFI, median (IQR)	394.5 (256, 934)	573 (336, 1418)	1018 (312, 1702)	**0.001**

Statistical significance was tested by the Kruskal-Wallis test,  ^$^Pearson Chi-Square test, and ^#^ordinary One-way ANOVA; The *P* value < 0.05 (bold numbers) indicates an overall significant difference among non-smokers, cigarette smokers, and e-cigarette users

*IQR* Interquartile Range, *BMI* Body Mass Index, *FEV*_*1*_ Forced Expiratory Volume in one second, *VC* Vital Capacity, *% pred* percentage predicted, *FeNO* Fractional exhaled nitric oxide, *SD* Standard Deviation, *IL* Interleukin, *TLR* Toll-like Receptors, *MFI* Median Fluorescence Intensity

^§^Data from Sompa, S. I., Ji, J., Rahman, M., Sjögren, B., Upadhyay, S., Ganguly, K., Olin, A.-C., Bergström, A., & Palmberg, L. (2025) [6]. Local and systemic effects in e-cigarette users compared to cigarette smokers, dual users, and non-smokers. *Respiratory Research 2025 26:1*, *26*(1), 1–15. 10.1186/S12931-025-03289-4

### Percentage of the lipid classes of the total lipid signal

A total of 9 lipid classes were identified in PEx samples. The molecular percentages of each lipid class of the total lipid signal are presented in Table 2. Among phospholipid classes, phosphatidylcholine (PC) accounted for 80% of the total lipid signal, being the most abundant phospholipid class in the PEx samples in all groups, followed by phosphatidylglycerol (PG), accounting for 9% of the total lipid signal. Other lipid classes contributed to less than 4% of the total lipid signal. The percentage of glucosylceramide (GlcCer), lysophosphatidylcholine (LPC), and phosphatidylcholine (PC) lipid classes of the total lipid signal was significantly different between healthy non-smokers, cigarette smokers, and e-cigarette users. However, the percentages of LPC, sphingomyelin (SM), and phosphotidylethanolamine (PE) lipid classes of the total lipid signal were significantly higher in e-cigarette users when compared to non-smokers (Table 2).

**Table 2:** The molecular percentage of each lipid class of the total lipid signal determined in the PEx samples

Lipid classes	Total lipid signal, %, median (IQR)			*P* value
	Non-smokers	Cigarette smokers	E-cigarette users	
GlcCer	0.01 (0.01, 0.02)	0.01 (0.01, 0.01)	0.04 (0.01, 0.06)	**0.02**
LPC	0.25 (0.2, 0.33)	0.22 (0.18, 0.3)	0.58 (0.18, 5.14)*†	**0.007**
SM	0.20 (0.16, 0.22)	0.20 (0.17, 0.25)	0.38 (0.16, 0.97)*†	0.05
PS	0.83 (0.76, 0.95)	0.77 (0.71, 0.94)	0.87 (0.78, 0.96)	0.3
PE	1.25 (1.17, 1.37)	1.27 (1.07, 1.41)	1.36 (1.23, 2.83)*†	0.05
PEO	2.11 (1.77, 2.38)	1.95 (1.46, 2.28)	1.95 (1.76, 2.51)	0.4
PI	3.87 (3.27, 4.25)	3.59 (2.72, 4.13)	3.15 (2.55, 4.01)	0.5
PG	9.91 (9.22, 10.3)	9.42 (8.3, 9.95)	8.96 (7.96, 9.90)	0.1
PC	81.8 (80.7, 82. 6)	82.1 (81.1, 83.6)	80.4 (74.3, 81.5)	**0.03**

Statistical significance was tested by the Kruskal-Wallis test followed by the Mann-Whitney test. The *P* value indicates comparisons between groups. *P* value < 0.05 (bold numbers) indicates an overall significant difference among non-smokers, cigarette smokers, and e-cigarette users

*Indicate* P* value < 0.05 compared with non-smokers (Mann-Whitney, unadjusted)

†Indicates *P* value < 0.05 compared with non-smokers adjusted for multiple comparisons by Dunn’s post hoc test

*GlcCer* Glucosylceramide, *LPC* Lysophosphatidylcholine, *SM* Sphingomyelin, *PS* Phosphatidylserine, *PE* Phosphatidylethanolamine, *PEO* Phosphatidylethanol Oxazoline, *PI* Phosphatidylinositol, *PG* Phosphatidylglycerol, *PC* Phosphatidylcholine, *IQR* Interquartile Range

### Lipid classes that separated e-cigarette users, cigarette smokers, and healthy non-smokers

**Fig. 1:**
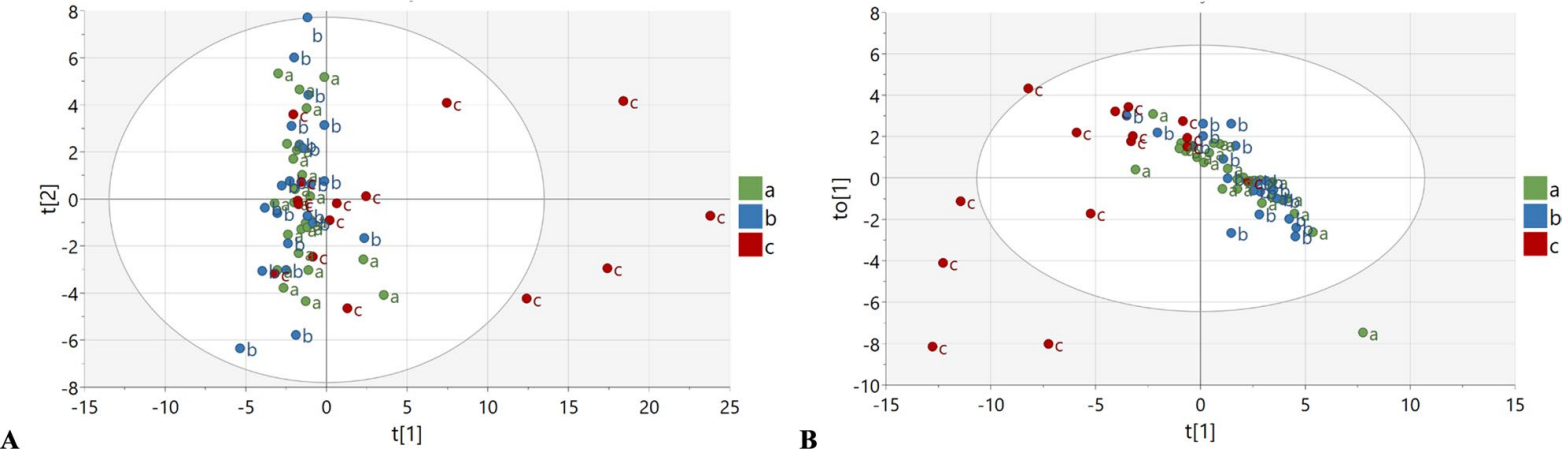
**A **The score plot from the first two components (t) of an unsupervised PCA model (R2X=0.80, Q2=0.38, at 9 components). Here, the score plot shows that five e-cigarette smokers are separated from the rest of the population. **B **The predictive (t) vs the orthogonal (to) scores from a supervised OPLS-DA model expanded to two components for the score plot (R2X=0.32, Q2=0.13, at 1 component). The score plots show group separation between healthy non-smokers (green, a) and cigarette smokers (blue, b) to the right versus e-cigarette smokers (red, c) to the left based on variations in individual lipid species variables. In this three-group comparison, no separation was obtained between non-smokers and cigarette smokers. The dots in the score plots represent individual PEx samples dependent on their phospholipid compositions. Closer dots indicate more similar compositions

The score plot for the nine different lipid classes expressed as individual lipid species from the unsupervised PCA model (Fig. 1A, R2X = 0.80, Q2 = 0.38) did not show any clear separation between groups for the lipid classes. The PCA plot shows, however, that the variation within the e-cigarette group is dominating the variation in the first component (Fig. 1A). While the lipid profiles of some e-cigarette users show overlap with cigarette smokers and non-smokers, there are at least five subjects located in the two right quadrants of the plot, indicating a marked difference in their underlying phospholipid profiles for those individuals compared to the other groups.

The score plot from the supervised OPLS-DA models, including all three groups in the same model (Fig. 1B), showed a distinct clustering of e-cigarette users from the other two groups for the lipid classes, with some overlap between groups. Although the optimal model was obtained for one component (R2X = 0.32, Q2 = 0.13), two components were used in Fig. 1B to better illustrate the group separations. No separation between cigarette smokers and non-smokers was shown by this three-group model.

### Lipid classes associated with e-cigarette users and cigarette smokers

**Fig. 2:**
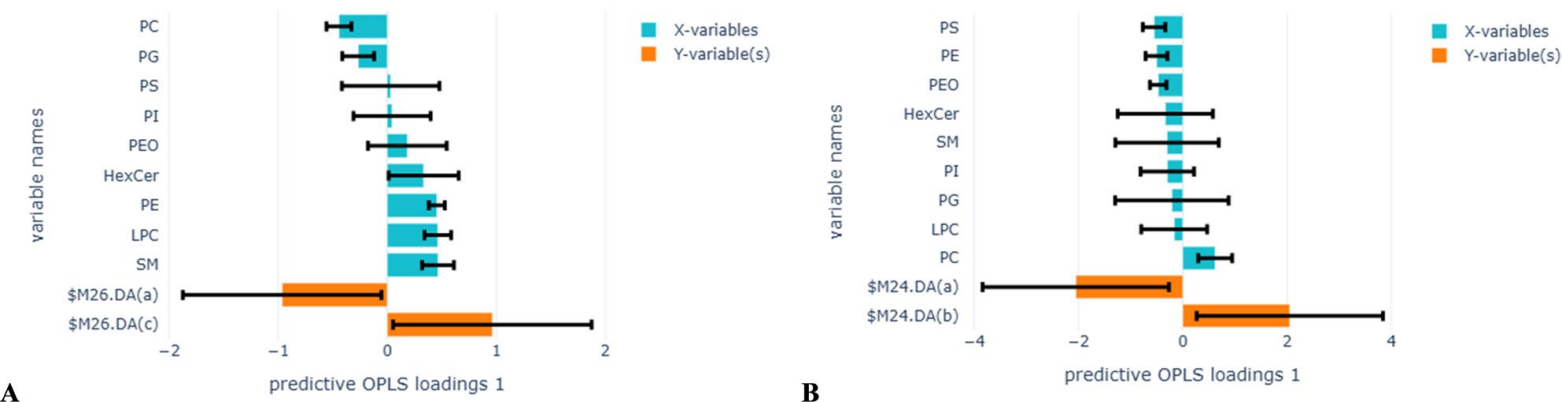
OPLS-DA analysis to identify lipid classes associated with e-cigarette users compared to non-smokers (**A**) (R2X=0.53, Q2=0.19, 1 component), and cigarette smokers compared to non-smokers (**B**) (R2X=0.37, Q2=-0.02, 1 component). Yellow bars in the positive direction represent e-cigarette users in figure **A** and cigarette smokers in figure **B**, and yellow bars in the negative direction represent non-smokers in both figures. Blue bars on the Y-axis represent lipid species. Error bars show the confidence intervals of the OPLS-DA loadings plot. Both significant and non-significant loadings are plotted

The OPLS-DA analysis operating as discrimination between two groups at the time showed a significant variation in the lipid classes to differentiate between e-cigarette users, cigarette smokers, and non-smokers (Fig. 2). In total, 5 lipid classes contributed to the model’s ability to differentiate between e-cigarette users and non-smokers (Fig. 2A). Among these lipid classes, higher levels of SM, LPC, and PE were strongly associated with e-cigarette users, and higher levels of PC and PG were strongly associated with non-smokers, even though the models were weak (Fig. 2A, R2X = 0.53, Q2 = 0.19). The models set out to identify the difference between cigarette smokers and non-smokers did not achieve the desired separation (Fig. 2B, R2X = 0.37, Q2=-0.02).

Linear regression analysis investigating the difference in the proportions of lipid classes between groups revealed that e-cigarette users had higher proportions of GlcCer, LPC, SM, and PE classes, and lower proportions of PC classes (Table 3), using non-smokers as a reference group. However, there were no significant differences in the proportions of lipid classes among cigarette smokers compared to non-smokers (Table 3).

**Table 3:** The difference in molecular percentage of each lipid class among e-cigarette users and cigarette smokers, analysed by linear regression with non-smokers as the reference group

Lipid class	Cigarette smokers			E-cigarette users		
	β (95% CI)	*P* value	Q value	β (95% CI)	*P* value	Q value
GlcCer	-0.005 (-0.02, 0.01)	0.5	0.7	0.03 (0.01, 0.05)	**0.005**	**0.02**
LPC	0.11 (-1.03, 1.26)	0.8	1	2.64 (1.37, 3.90)	**< 0.001**	**0.0004**
SM	-0.004 (-0.17, 0.16)	0.9	1	0.39 (0.20, 0.57)	**< 0.001**	**0.0003**
PS	-0.06 (− 0.15, 0.02)	0.1	0.3	− 0.002 (− 0.10, 0.09)	0.9	1
PE	-0.01 (-0.45, 0.42)	0.9	1	0.95 (0.46, 1.44)	**< 0.001**	**0.0004**
PEO	-0.28 (-0.66, 0.09)	0.1	0.2	0.04 (-0.37, 0.45)	0.8	1
PI	-0.10 (-0.76, 0.54)	0.7	0.9	-0.23 (-0.95, 0.48)	0.5	1
PG	-0.49 (-1.35, 0.36)	0.2	0.3	-0.75 (-1.70, 0.18)	0.1	0.2
PC	0.86 (-1.0, 2.73)	0.3	0.4	-3.06 (-5.12, -1.0)	**0.004**	**0.008**

*P* value < 0.05 (bold numbers) indicates significant associations compared with non-smokers; Q value: *P* value adjusted for False Discovery Rate (FDR) by the Benjamini-Hochberg (BH) procedure

*β* Regression coefficient, *95% CI* 95% Confidence interval

### Lipid species associated with e-cigarette users and cigarette smokers

**Fig. 3:**
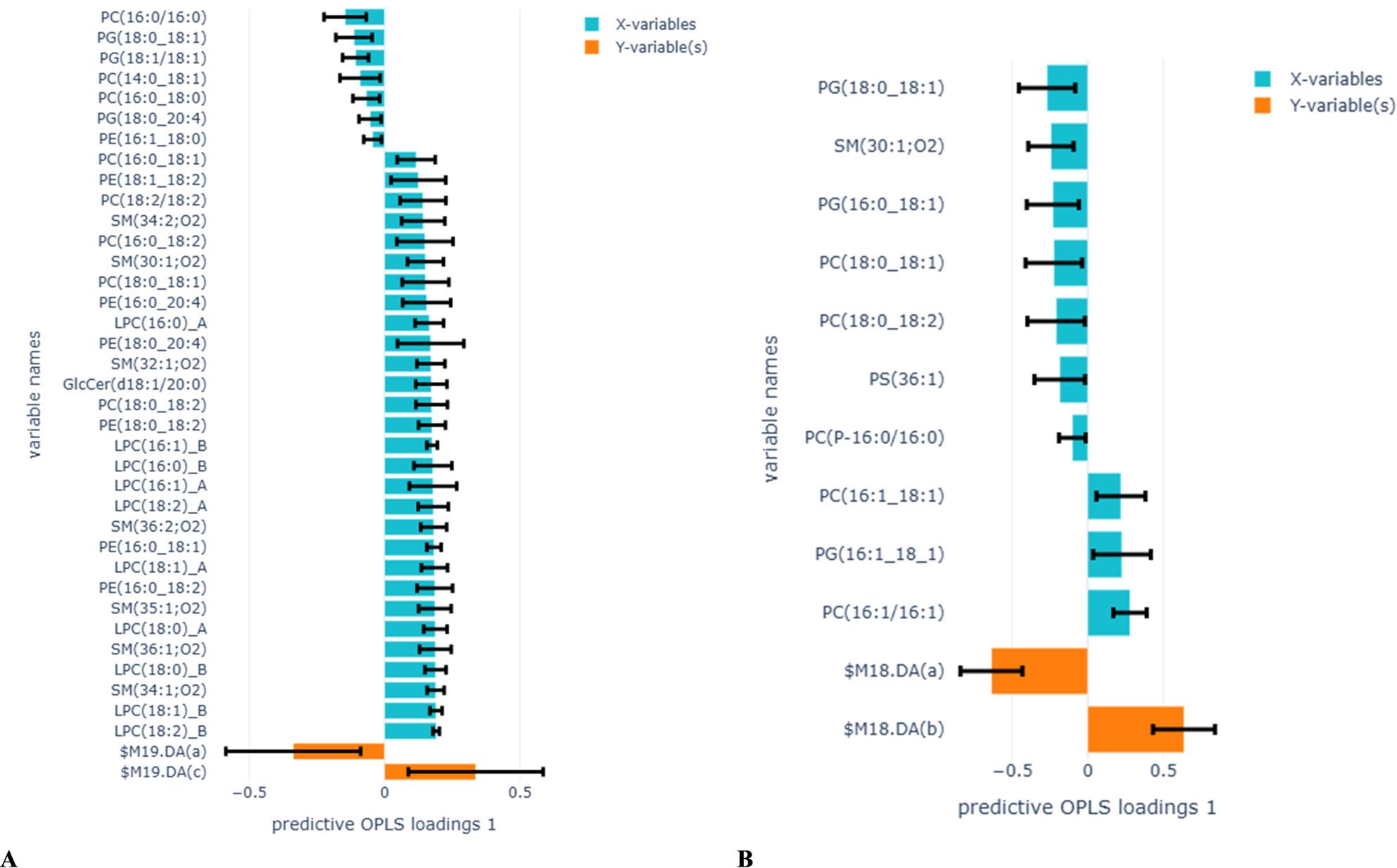
OPLS-DA analysis to identify lipid species associated with e-cigarette users compared to non-smokers (**A**) (R2X=0.35, Q2=0.23, 1 component), and cigarette smokers compared to non-smokers (**B**) (R2X=0.06, Q2=0.02, 2 components). Yellow bars in the positive direction represent e-cigarette users in figure **A** and cigarette smokers in figure **B**, and yellow bars in the negative direction represent non-smokers in both figures. Blue bars on the Y-axis represent lipid species. Error bars show the confidence intervals of the OPLS-DA loadings plot. Only significant loadings are plotted

In total, 86 lipid species (Table S1) belonging to the 9 lipid classes were identified in the PEx samples. Of these, 36 lipid species significantly impacted the OPLS model’s ability to differentiate between e-cigarette users and non-smokers (Fig. 3A). Among lipid species, higher levels of LPC(18:2)_B showed the strongest association with e-cigarette users, and PC(16:0/16:0) with non-smokers. Higher levels of LPC(18:1)_B, LPC(18:1)_A, LPC(18:0)_B, LPC(18:0)_A, LPC(16:1)_A, LPC(16:1)_B LPC(16:0)_B, SM(34:1:02), SM(36:1:01), SM(35:1:02), SM(32:1;O2), PE(16:0_18:2), PE(16:0_18:1), PC(18:0/18:2), and GlcCer(d18:1/20:0) were also strongly associated with e-cigarette users. The levels of PC(16:0/16:0), PG(18:0_18:1), and PG(18:1_18:1) were higher and strongly associated with non-smokers and were significant in the model. In addition, 10 phospholipid species significantly contributed to the model’s ability to discriminate between cigarette smokers and non-smokers (Fig. 3B). Of these, higher levels of PC(16:1/16:1) were strongly associated with cigarette smokers, and PG(18:0_18:1) with non-smokers.

Linear regression analysis investigating the difference in the proportions of individual lipid species between groups showed that e-cigarette users had higher proportions of 34 phospholipid species and lower proportions of 4 phospholipid species compared to non-smokers (Table 4). Of these phospholipid species, LPC(18:0)_A, LPC(18:1)_A, LPC(18:1)_B, LPC(18:2)_A, LPC(18:2)_B, PE(16:0_18:1), PE(16:0_18:2), PE(18:0_18:1), PE(18:0_18:2), SM(32:1;O2), SM(34:1;O2), SM(35:1;O2), SM(36:1;O2), SM(36:2;O2), PC(16:0_18:2), and PC(18:0_18:2) were higher and exhibited the lowest *p* value of < 0.001 (Table 4). On the other hand, cigarette smokers had higher proportions of 2 phospholipid species and lower proportions of 4 phospholipid species compared to non-smokers in the linear regression analysis (Table 5). Of these, phospholipid species PC(16:1/16:1) were higher, and PG(18:0_18:1) and PS(36:1) were lower, and exhibited the lowest *p* value of 0.01 (Table 5).

**Table 4:** The difference in molecular percentage of each lipid species among e-cigarette users, analysed by linear regression with non-smokers as the reference group. Only significant data are shown

Lipid species	E-cigarette users		
	β (95% CI)	*P* value	Q value
GlcCer(d18:1/16:0)	0.02 (0.006, 0.03)	**0.005**	**0.02**
GlcCer(d18:1/18:0)	0.005 (0.0009, 0.01)	**0.01**	**0.07**
GlcCer(d18:1/20:0)	0.001 (0.0005, 0.02)	**0.003**	**0.006**
GlcCer(d18:1/22:0)	0.006 (0.002, 0.01)	**0.005**	**0.009**
LPC(16:0)_A	0.06 (0.01, 0.1)	**0.02**	**0.03**
LPC(16:0)_B	0.4 (0.1, 0.6)	**0.001**	**0.003**
LPC(16:1)_A	0.01 (0.004, 0.01)	**0.001**	**0.003**
LPC(16:1)_B	0.01 (0.005, 0.02)	**0.001**	**0.003**
LPC(18:0)_A	0.05 (0.02, 0.08)	**< 0.001**	**0.001**
LPC(18:0)_B	0.1 (0.06, 0.2)	**0.001**	**0.005**
LPC(18:1)_A	0.9 (0.4, 1.3)	**< 0.001**	**0.0006**
LPC(18:1)_B	0.2 (0.1, 0.3)	**< 0.001**	**0.0002**
LPC(18:2)_A	0.6 (0.3, 0.9)	**< 0.001**	**0.001**
LPC(18:2)_B	0.1 (0.08, 0.2)	**< 0.001**	**0.0002**
SM(30:1;O2)	0.0008 (0.0002, 0.001)	**0.005**	**0.009**
SM(32:1;O2)	0.01 (0.009, 0.02)	**< 0.001**	**0.0004**
SM(34:1;O2)	0.3 (0.1, 0.4)	**< 0.001**	**0.0004**
SM(34:2;O2)	0.002 (0.0005, 0.004)	**0.01**	**0.03**
SM(35:1;O2)	0.01 (0.009, 0.02)	**< 0.001**	**0.00001**
SM(36:1;O2)	0.04 (0.2, 0.06)	**< 0.001**	**0.0002**
SM(36:2;O2)	0.003 (0.001, 0.005)	**< 0.001**	**0.0004**
PE(16:0_18:1)	0.2 (0.1, 0.3)	**< 0.001**	**0.0003**
PE(16:0_18:2)	0.2 (0.1, 0.3)	**< 0.001**	**0.0002**
PE(16:0_20:4)	0.02 (0.01, 0.04)	**0.001**	**0.002**
PE(18:0_18:1)	0.1 (0.05, 0.1)	**< 0.001**	**0.0003**
PE(18:0_18:2)	0.1 (0.06, 0.2)	**< 0.001**	**0.001**
PE(18:0_20:4)	0.1 (0.04, 0.1)	**0.001**	**0.003**
PE(18:1_18:2)	0.05 (0.01, 0.09)	**0.009**	**0.01**
PG(16:0_18:2)	0.04 (0.004, 0.07)	**0.02**	**0.05**
PG(18:0_18:1)	-0.3 (-0.5, -0.1)	**0.004**	**0.008**
PC(14:0_18:1)	-0.09 (-0.18, -0.006)	**0.03**	**0.07**
PC(16:0_17:0)	-0.2 (-0.3, -0.07)	**0.004**	**0.007**
PC(16:0_18:2)	0.3 (0.1, 0.5)	**< 0.001**	**0.0008**
PC(16:1_18:0)	-0.03 (-0.07, -0.002)	**0.03**	**0.06**
PC(18:0_18:1)	0.07 (0.0005, 0.1)	**0.04**	**0.09**
PC(18:0_18:2)	0.4 (0.1, 0.6)	**< 0.001**	**0.0009**
PC(18:1_20:4)	0.05 (0.01, 0.09)	**0.006**	**0.02**
PC(18:2/18:2)	0.1 (0.06, 0.2)	**0.001**	**0.002**

*P* value < 0.05 (bold numbers) indicates significant associations; Q value: *P* value adjusted for False Discovery Rate (FDR) by the Benjamini-Hochberg (BH) procedure

*β* Regression coefficient, *95% CI* 95% Confidence interval

**Table 5:** The difference in molecular percentage of each lipid species among cigarette smokers, analysed by linear regression with non-smokers as the reference group. Only significant data are shown

Lipid species	Cigarette smokers		
	β (95% CI)	*P* value	Q value
PG(16:0_18:1)	-0.3 (-0.6, -0.05)	**0.02**	**0.01**
PG(18:0_18:1)	-0.2 (-0.4, -0.06)	**0.01**	**0.04**
PS(36:1)	-0.06 (-0.1, -0.01)	**0.01**	**0.03**
SM(30:1;O2)	-0.0005 (-0.001, -0.00001)	**0.04**	**0.05**
PC(16:1/16:1)	0.04 (0.009, 0.07)	**0.01**	**0.02**
PC(16:1_18:1)	0.05 (0.001, 0.1)	**0.04**	**0.09**

*P* value < 0.05 (bold numbers) indicates significant associations; Q value: *P* value adjusted for False Discovery Rate (FDR) by the Benjamini-Hochberg (BH) procedure

*β* Regression coefficient, *95% CI* 95% Confidence interval

Notably, linear regression analysis showed that both e-cigarette users and cigarette users had significantly lower proportions of phosphatidylglycerol species PG(18:0_18:1) (Tables 4 and 5). In addition, e-cigarette users had higher proportions (Table 4), and cigarette smokers had lower proportions (Table 5) of sphingomyelin species SM(30:1;O2).

### Lipid classes and lipid species in association with IL-13-producing T cells in blood, FeNO, and surface TLR2 expression in sputum in e-cigarette users and cigarette smokers

Linear regression analysis showed that IL-13-producing T cells in blood were significantly associated with higher proportions of phospholipid classes LPC, SM, and PE, and with lower proportions of PC in e-cigarette users compared to non-smokers (Table 6). FeNO was also significantly associated with higher levels of phospholipid class PG in e-cigarette users compared to non-smokers (Table 6). However, no significant results were observed between TLR2 in sputum and lipid classes in e-cigarette users (Table 6). In addition, there was no significant association of FeNO, IL13-producing T cells in blood, and TLR2 in sputum with lipid classes in cigarette smokers in linear regression analysis (results not shown).

**Table 6:** The association of lipid classes with IL-13-producing T cells in blood, FeNO, and TLR2 in sputum immune cells among e-cigarette users, analysed by linear regression with interaction terms, compared with non-smokers as the reference group

Lipid class	IL-13-producing T cells in the blood			FeNO			TLR2 in sputum immune cells		
	β (95% CI)	*P* value	Q value	β (95% CI)	*P* value	Q value	β (95% CI)	*P* value	Q value
GlcCer	0.0004 (-0.0004, 0.001)	0.2	0.7	-0.002 (-0.005, 0.001)	0.2	0.9	-0.000006 (-0.00006, 0.00005)	0.8	1
LPC	0.07 (0.03, 0.1)	**< 0.001**	**0.003**	-0.1 (-0.3, 0.04)	0.1	0.5	-0.001 (-0.004, 0.0007)	0.1	0.6
SM	0.009 (0.003, 0.01)	**0.002**	**0.007**	-0.02 (-0.04, 0.005)	0.1	0.4	-0.0002 (-0.0006, 0.00006)	0.1	0.2
PS	0.001 (-0.003, 0.005)	0.5	0.8	0.001 (-0.01, 0.01)	0.8	1	-0.0001 (-0.0004, 0.00008)	0.1	0.4
PE	0.02 (0.009, 0.04)	**0.003**	**0.01**	-0.04 (-0.1, 0.02)	0.2	0.6	-0.0009 (-0.001, 0.0001)	0.07	0.2
PEO	0.01 (-0.003, 0.03)	0.1	0.4	-0.05 (-0.1, 0.01)	0.1	0.4	-0.0009 (-0.0008, 0.00006)	0.06	0.1
PI	-0.006 (-0.03, 0.02)	0.6	1	-0.03 (-0.1, 0.07)	0.5	1	0.0001 (-0.001, 0.001)	0.9	1
PG	-0.03 (-0.07, 0.01)	0.1	0.5	0.1 (0.02, 0.2)	**0.02**	0.05	0.0007 (-0.001, 0.002)	0.4	1
PC	-0.09 (-0.1, -0.01)	**0.01**	0.06	0.1 (-0.1, 0.4)	0.3	1	0.003 (-0.001, 0.008)	0.1	0.4

*P* value < 0.05 (bold numbers) indicates a significant association; Q value: *P* value adjusted for False Discovery Rate (FDR) by the Benjamini-Hochberg (BH) procedure

*β* Regression coefficient, *95% CI* 95% Confidence interval

**Fig. 4:**
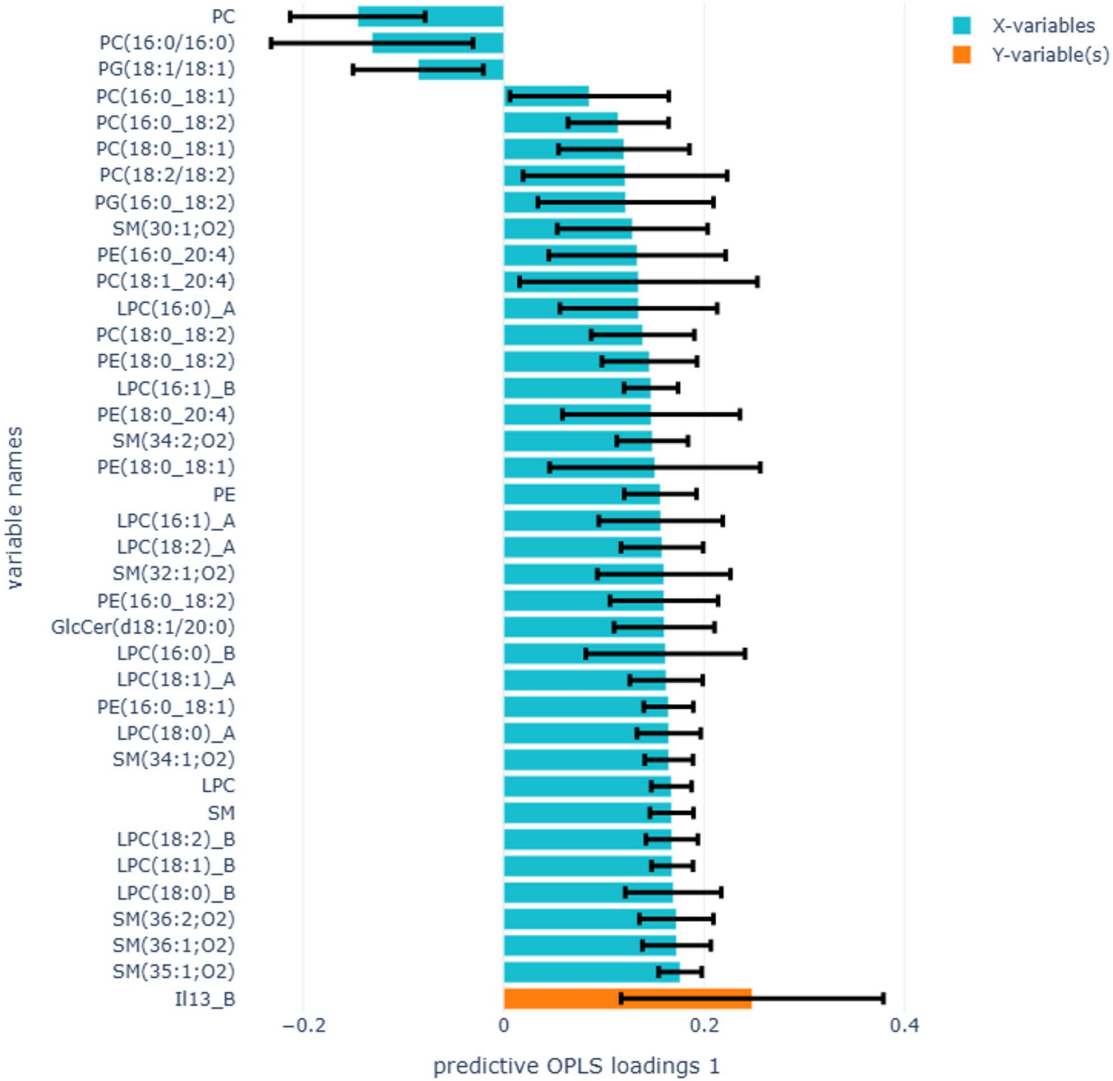
OPLS regression analysis to identify lipid species associated with IL-13 in blood in e-cigarette users compared to non-smokers (R2X=0.45, Q2=0.41, 2 components). The yellow bar represents IL-13, and the blue bars represent lipid species. Error bars show the confidence intervals of the loadings plot, which are significant when they do not include zero. Only significant loadings are plotted

**Fig. 5:**
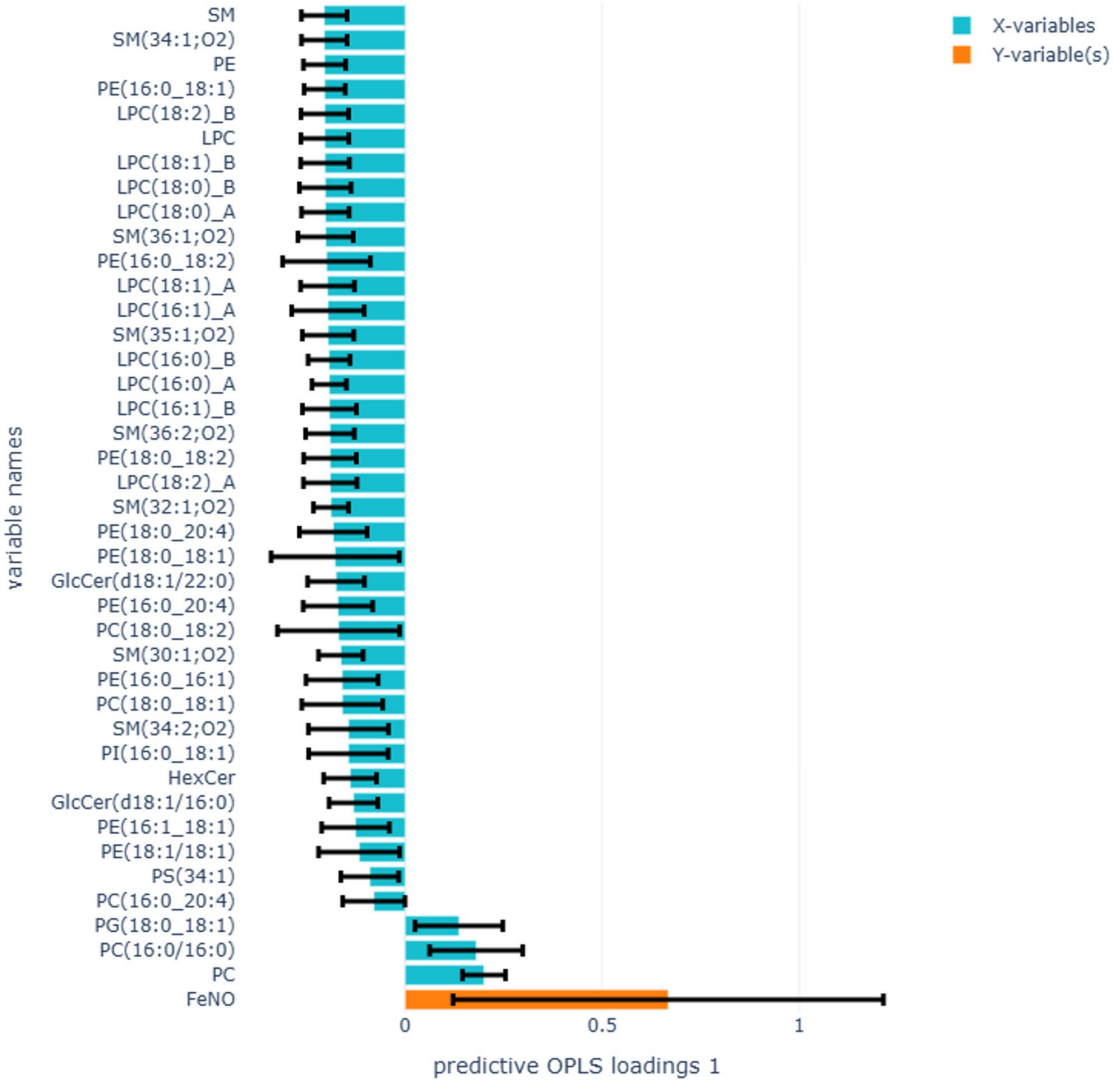
OPLS-DA analysis to identify lipid species associated with FeNO in e-cigarette users compared to non-smokers (R2X=0.34, Q2=-0.05, 2 components). The yellow bar represents FeNO, and the blue bars represent lipid species. Error bars show the confidence intervals of the loadings plot, which are significant when they do not include zero. Only significant loadings are plotted

**Fig. 6:**
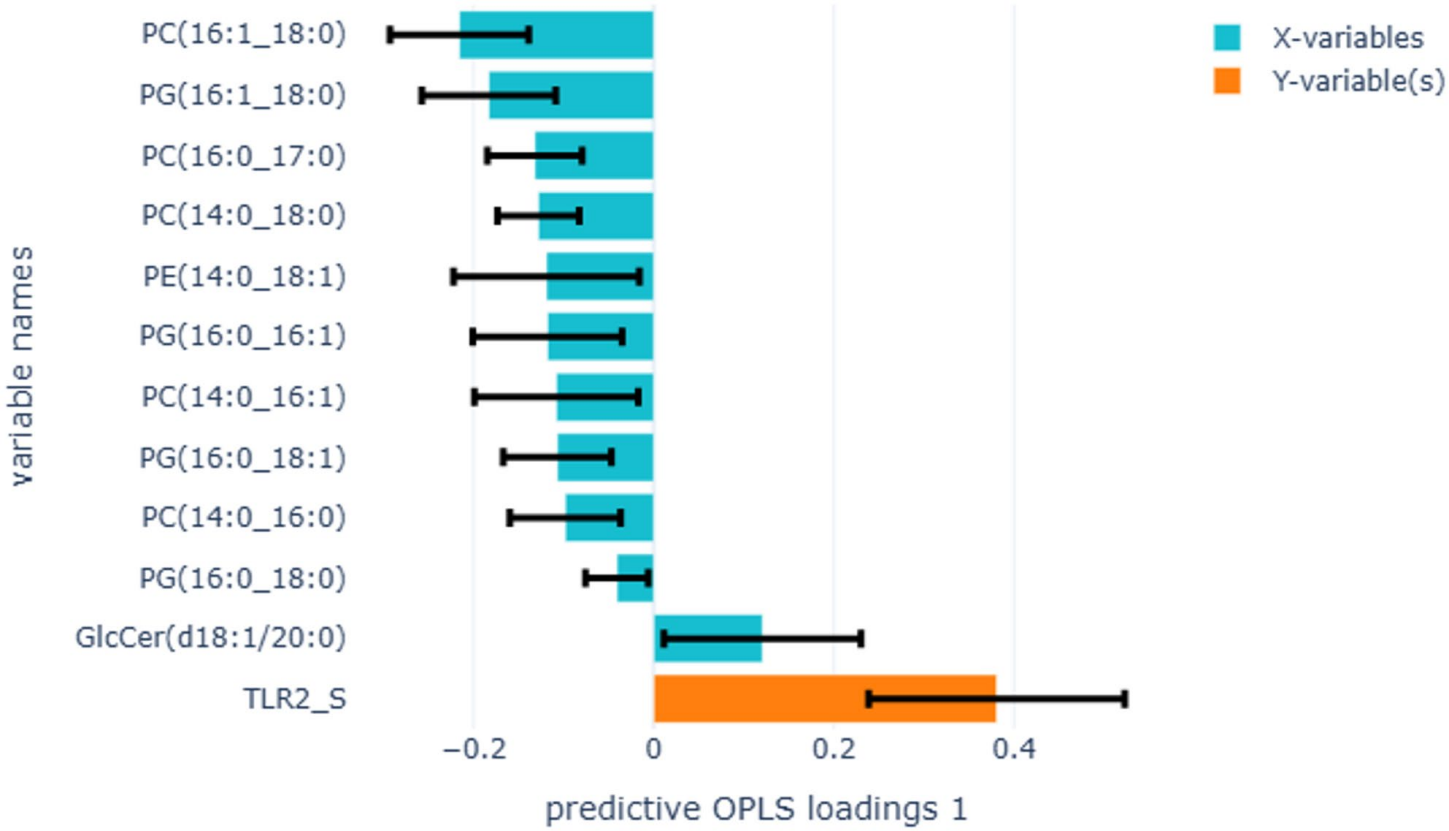
OPLS-DA analysis to identify lipid species associated with TLR2 in sputum immune cells in e-cigarette users compared to non-smokers (R2X=0.12, Q2=0.04, 2 components). The yellow bar represents TLR2, and the blue bars represent lipid species. Error bars show the confidence intervals of the loadings plot, which are significant when they do not include zero. Only significant loadings are plotted

OPLS regression analysing differences between IL-13-producing T cells in blood and proportions of phospholipid species belonging to the LPC, SM, PE, and PC classes in e-cigarette users compared to non-smokers produced a stronger model (Fig. 4 R2X = 0.45, Q2 = 0.41), and the results were in accordance with the linear regression models (Table 7). Similar OPLS analysis between the proportions of lipid species and FeNO (Fig. 5, R2X = 0.35, Q2 = -0.06, 1 component) and TLR2 in sputum (Fig. 6, R2X = 0.47, Q2 = 0.04, 2 components) in e-cigarette users compared to non-smokers produced weak models. Analysing differences between the proportion of lipid profiles and IL-13-producing T cells in blood, FeNO, and TLR2 in sputum with lipid classes in cigarette smokers compared to non-smokers also produced weak models (results not shown).

**Table 7:** The association of lipid species with IL-13-producing T cells in blood among e-cigarette users, analysed by linear regression with interaction terms, compared with non-smokers as the reference group. Only significant data are shown

Lipid species	IL-13-producing T cells in the blood		
	β (95% CI)	*P* value	Q value
LPC(16:0)_A	0.002 (0.0001, 0.004)	**0.04**	0.1
LPC(16:1)_A	0.0003 (0.00008, 0.0005)	**0.008**	0.06
LPC(16:1)_B	0.0004 (0.0004, 0.0007)	**0.002**	**0.01**
LPC(18:0)_A	0.0015 (0.0004, 0.002)	**0.006**	**0.04**
LPC(18:1)_A	0.02 (0.01, 0.04)	**0.001**	**0.009**
LPC(18:1)_B	0.0057 (0.002, 0.009)	**0.001**	**0.009**
LPC(18:2)_A	0.01 (0.007, 0.03)	**0.002**	**0.01**
LPC(18:2)_B	0.004 (0.001, 0.006)	**0.002**	**0.01**
SM(34:1;O2)	0.007 (0.002, 0.012)	**0.005**	**0.02**
SM(35:1;O2)	0.0003 (0.0001, 0.0005)	**0.001**	**0.01**
SM(36:1;O2)	0.0009 (0.0002, 0.001)	**0.006**	**0.02**
PE(16:0_18:1)	0.006 (0.001, 0.01)	**0.006**	**0.02**
PE(16:0_18:2)	0.006 (0.002, 0.01)	**0.004**	**0.03**
PE(18:0_18:1)	0.002 (0.0007, 0.004)	**0.007**	**0.02**
PE(18:0_18:2)	0.003 (0.001, 0.006)	**0.007**	**0.02**
PE(18:0_20:4)	0.002 (0.0006, 0.005)	**0.01**	0.09
PC(16:0_18:1)	0.04 (0.01, 0.07)	**0.01**	**0.02**
PC(16:0_18:2)	0.01 (0.004, 0.02)	**0.005**	**0.01**
PC(18:0_18:1)	0.004 (0.001, 0.006)	**0.002**	**0.001**
PC(18:0_18:2)	0.01 (0.004, 0.02)	**0.004**	**0.01**

*P* value < 0.05 (bold numbers) indicates significant associations; Q value: *P* value adjusted for False Discovery Rate (FDR) by the Benjamini-Hochberg (BH) procedure

*β* Regression coefficient, *95% CI* 95% Confidence interval

## Discussion

This study is the first to examine the lipid profile in the lining fluid of small airways in e-cigarette users compared to cigarette smokers and non-smokers. The analysis revealed significant differences in both lipid classes and individual lipid species between e-cigarette users, cigarette smokers, and non-smokers.

Interestingly, the differences in the lipid profile in e-cigarette users compared to non-smokers were much stronger than between cigarette smokers and non-smokers. Another interesting finding was the strong association found between IL-13-producing T cells in blood and lipid profile in e-cigarette users compared to non-smokers. This suggests that the profile of lipid species is associated with inflammatory pathways induced by the use of e-cigarettes. Indeed, many of the lipid species that were higher among e-cigarette users in our study, such as PC(18:0/18:1), PE(18:0/20:4), and PE(16:0/18:2), have been found to be associated with inflammation [31]. This gives rise to concerns, also indicated by our previous study, where the use of e-cigarettes was found to induce different markers of local and systemic inflammatory responses [6].

### Differences in lipids in response to e-cigarettes and traditional cigarettes

PC is the major lipid mediator found in the lung surfactant, which is crucial for maintaining normal lung function. Decreased levels of PC may impair surface tension and are associated with various lung diseases, including ARDS, interstitial lung diseases, and pneumonia [18, 32]. Notably, PC(16:0/16:0) is the main component of the PC lipid class and is responsible for effectively reducing surface tension, indicating its importance in maintaining airway potency. Additionally, other PC species such as PC(16:0/14:0) and PC(16:0/16:1) have been reported to be associated with respiratory rate in mammals [32]. In our study, while the total percentage of PC lipid class did not differ significantly among e-cigarette users compared to non-smokers, we identified a significantly lower proportion of certain PC species, such as PC(16:1/16:1), significantly higher proportion of PC(16:0_18:2) and PC(18:0/18:2), but no difference in the proportions of PC(16:0/ 16:0). The proportion of PC(16:1/16:1) was increased in cigarette smokers compared to non-smokers. The clinical importance of these findings is not known, but the increased levels of polyunsaturated PC species are suggested to influence surfactant function [33], increasing surface tension.

LPC is mainly produced by the hydrolysis of PC and is known for mediating oxidative stress and inflammatory response [34, 35]. Pro-inflammatory activities of LPC are mediated in different ways, such as upregulated gene expression in endothelial cells, increased release of cytokines (IL-1β, IL-6, and TNF-α) from adipocytes, and enhanced activation of B cells and macrophages. Additionally, LPC promotes the function of regulatory T cells, which suppress immune response through anti-inflammatory cytokine production [35]. In our study, we observed a significantly higher molecular percentage of LPC in e-cigarette users, corresponding to a 2.5-fold relative increase compared to non-smokers. This is comparable to a 2- and 1.3-fold relative increase of LPC(18:0) and LPC(16:0) molecules in the BAL fluid of asthma patients who had impaired lung function compared to non-asthmatic controls [34]. This could suggest that even modest changes in lipid composition may be associated with variations in airway immune or inflammatory conditions. Linear regression analysis further revealed significant differences in the proportions of LPC class and individual LPC lipid species among e-cigarette users compared to healthy non-smokers. It is known that saturated LPCs like LPC (16:0) induce inflammation, promoting immune cell migration and pro-inflammatory factor release. Conversely, polyunsaturated LPCs such as LPC (20:4), LPC (20:5), and LPC (22:6) act as anti-inflammatory agents, countering saturated LPCs’ immune response [36]. In addition, LPC14:0, although a saturated LPC, showed anti-inflammatory and anti-oxidative stress effects both in vivo and in vitro experiments in a previous study [37]. Higher levels of plasma LPC(14:0) were also found to be associated with a better prognosis in community-acquired pneumonia [37]. These findings suggest the role of LPC as both potential biomarkers and therapeutic targets in inflammatory lung diseases. In the current study, we identified that the proportions of LPC(18:0)_A and LPC(16:0)_B, along with other LPC species such as LPC(18:2), LPC(18:1), and LPC(16:1), were higher in e-cigarette users. Since the physiological role of LPC is complex and knowledge is still limited, the observed LPC differences in our study should be interpreted with caution rather than as definitive evidence of causal effects or direct physiological changes.

SM is mainly present in the cell membrane and is essential for normal lung physiology [38]. In pulmonary surfactant, the levels of SM are normally very low [10], but have been reported to contribute to pulmonary surfactant composition and alveolar stability [38]. Disruptions in the metabolism of SM are involved in lung diseases such as chronic obstructive pulmonary disease (COPD) and cystic fibrosis [38]. In this study, the proportions of the lipids belonging to the SM class were significantly higher in e-cigarette users. In previous case-control studies, SM(t34:1) and SM(d35:1) extracted from frozen tissue samples were found to be potent predictors for lung adenocarcinoma recurrence after radical surgery [39, 40]. In another study, SM (d18:1/18:1) was reported to be significantly higher in asthma patients compared to healthy controls [41]. In our study, SM species such as SM(34:1:O2), SM(35:1:O2), and SM(36:1:O1) were significantly higher in e-cigarette use, and SM(30:1;O2) was higher in cigarette smokers compared to healthy non-smokers. These changes in the composition of the lining fluid with higher levels of SM species in e-cigarette users may indicate increased apoptosis and debris in the lining fluid.

PE is another phospholipid component of pulmonary surfactant [17], well-known for its role in oxidative stress, apoptosis, ferroptosis, and cell death in mammalian cells [42]. PE has been hypothesized as a potential biomarker in e-cigarette or vaping product-associated lung injury (EVALI) patients [43]. Oxidised PE has been reported to be associated with airway hyperresponsiveness in asthma patients [44]. Polyunsaturated PE is also associated with ferroptosis-inducing lipid peroxidation, i.e., programmed cell death [45–47]. In the current study, the proportion of PE was significantly higher in e-cigarette users, and the corresponding regression analysis found significantly higher proportions of both the PE class and specific PE species in e-cigarette users. In a previous study, PE (18:1p/22:6), PE (20:0/18:1), and PE (38:1) species were significantly higher in plasma samples from asthma patients compared to healthy controls [41]. In our current study, the proportions of PE(16:0_18:1), PE(16:0_18:2), PE(18:0_18:1), and PE(18:0_18:2) species were increased in e-cigarette users.

The mechanisms underlying the changes in airway lipid composition among e-cigarette users are not fully understood yet. Constituents of e-cigarette aerosol, including propylene glycol and various flavoring agents, may alter lung lipid homeostasis [48]. In a mouse model, exposure to chronic e-cigarette vapor altered lung lipid profiles, including the deposition of phospholipids in alveolar macrophages and changes in surfactant-associated lipids, independent of nicotine [48]. In humans, distinct changes in circulating lipid profiles have been observed in e-cigarette users compared with non-users and smokers, particularly in lipids relevant to surfactant function [49]. While the precise mechanisms linking e-cigarette aerosol components to specific airway lipid species such as LPC, PE, and SM remain to be fully elucidated, these studies suggest that aerosol-associated lipid disturbances in the lungs are plausible. Future research will be crucial in determining whether these subtle changes significantly impact airway physiology and immune function.

While the precise mechanisms connecting e-cigarette aerosol components to specific airway lipid species, such as lysophosphatidylcholine (LPC), phosphatidylethanolamine (PE), and sphingomyelin (SM), have not yet been fully clarified, these studies suggest that aerosol-associated lipid disturbances in the lungs are plausible. Future research will be crucial in determining whether these subtle changes significantly impact airway physiology and immune function.

### Association between lipids and inflammatory mediators in response to e-cigarettes

In this study, we found a significant association between IL-13-producing T cells in blood and increased levels of lipid classes LPC, SM, and PE among e-cigarette users in a linear regression model. The association between lipid species and IL-13 evaluated by the supervised OPLS-DA model exhibited a Q2 value of 0.41, and R2X = 0.45, indicating a good predictive ability, also confirmed in linear regression models of individual lipid species. The presence of IL-13 is a characteristic feature of Type Ⅱ inflammation in asthma. In addition, IL-13 induces NO production, hyperplasia, and metaplasia of mucous-producing goblet cells [50]. In our previous study, significantly higher percentages of IL-13-producing T cells in blood in e-cigarette users compared to non-smokers were found [6]. Recently, IL-13 has been found to influence lipid metabolism and to play a role in ferroptosis in asthmatic epithelial cells [51].

A significant association was also found between FeNO and increased PG lipid class in e-cigarette users from linear regression analysis. However, the corresponding OPLS-DA model was very weak, possibly due to large variation within the e-cigarette group, and a larger study is required to elucidate this further.

### Limitations and strengths

The study has several limitations. There was wide variation in the types of e-cigarettes used by participants, including differences in devices, e-liquid flavours, and nicotine content. With over fifteen hundred different flavours available in the market and varying nicotine levels [52], standardizing exposure assessment becomes complicated, making it difficult to interpret use patterns among small groups. Additionally, due to significant variability among users, standardizing exposure information into a single pack-year metric, commonly used for traditional cigarettes, is not feasible for e-cigarettes. Currently, there is no standardized equivalent measure, but combining self-reported usage patterns and duration provides the most practical exposure assessment, ensuring meaningful comparisons between cigarette smokers and e-cigarette users. Furthermore, the small sample size within each group of the study may limit the power of the investigated outcome, while strict inclusion criteria enhance the validity of the exposure outcome.

Another limitation of the study is its inability to distinguish between acute and chronic exposure effects, as participants varied widely in their frequency and duration of e-cigarette use (data not shown). In addition, strict inclusion criteria were implemented across all groups to reduce biological variability and limit confounding factors. Although this may have resulted in a healthier subset of cigarette smokers, the inclusion criteria are the same in all three groups, and it ultimately strengthens the study’s internal validity. Furthermore, the duration of exposure to nicotine products for cigarette smokers and e-cigarette user groups is about the same number of years, although it is not possible to match it totally, since it is measured differently. This implies that the observed differences are more likely attributable to the exposure to traditional cigarettes and e-cigarettes rather than underlying health conditions. It is essential to consider this limitation when interpreting the results. Moreover, the effects of e-cigarettes on lipid profiles appeared to differ more substantially than those from traditional cigarette smoke. This may suggest that the compounds delivered through e-cigarettes are less homogeneous, warranting further investigation into which specific compounds might explain these disparities. To assess the effect of smoking on up to 99 different lipid species, which are partly correlated to each other, also presents challenges. The knowledge of the biological function and role in the lining fluid of small airways of many of the different lipid species is still unknown.

However, a notable strength is that we got similar trends of results, using both regression analyses and multivariate analyses, where the former analyses one lipid at a time, introducing the problem of multiple testing, and the latter gives rise to multidimensional vectors, including all the complex mixture of lipids at the same time.

To assess the predictive performance of the PCA and OPLS models, the Q2 value was utilized from multivariate analysis. This value indicates the proportion of variance in the response variable that can be predicted by the model. For explorative studies, in general, values > 0.3 are suggested [53]. The unsupervised PCA model for lipid classes generated a cumulative Q2 value of 0.34 at 2 components, while the model based on individual lipid species had Q2 = 0.38 at 9 components, which reflects overall variation in lipid or lipid classes between groups and test persons. When focusing only on the variation between classes, the supervised OPLS-DA model between e-cigarette users, cigarette smokers, and non-smokers with only lipid class variables exhibited a good Q2 value of 0.19, still low, but better predictive ability for class separation than when having three classes in the same model. This result is merely indicative, given the explorative nature of the study, where this model only included 9 outcomes, and in all 62 subjects.

Overall, the models only containing the nine different lipid classes as predictors are much less robust than those containing all 99 lipid species, where many models achieved acceptable Q2-values. Especially for the model of the lipid species and IL-13-producing T cells in blood that achieved a Q2-value of 0.41, indicating a promising way for further research.

## Conclusions

Our study found significant differences in lipid profiles in the lining fluid of small airways among e-cigarette users compared to non-smokers. The differences were also associated with higher levels of IL-13-producing T cells among e-cigarette users compared to non-smokers. A higher proportion of these lipids indicates disturbances in lipid homeostasis and could potentially serve as early biomarkers in the development of chronic inflammatory lung diseases. This highlights the need for additional longitudinal studies on the long-term pulmonary effects of e-cigarette use, as the precise implications of our results remain unclear.

## Supplementary Information

Additional file 1: Table S1. A list of the phospholipid species identified in PEx samples.

Additional file 2: Figure S1. A flow chart illustrating the inclusion of the participants.

## Data Availability

The datasets used and/or analysed during the current study are available from the corresponding author on reasonable request.
