# Supplementary material for: Investigating the exhaled lipid biomarkers among e-cigarette users compared to non-smokers
Source: Lipids Health Dis. 2026 Feb 27;25:102. doi: 10.1186/s12944-026-02911-8 (PMC13049796; doi:10.1186/s12944-026-02911-8)
Supplement: Supplementary file 2 — Additional file 2: Figure S1. A flow chart illustrating the inclusion of the participants. [file 12944_2026_2911_MOESM2_ESM.docx]

**Supplementary data**


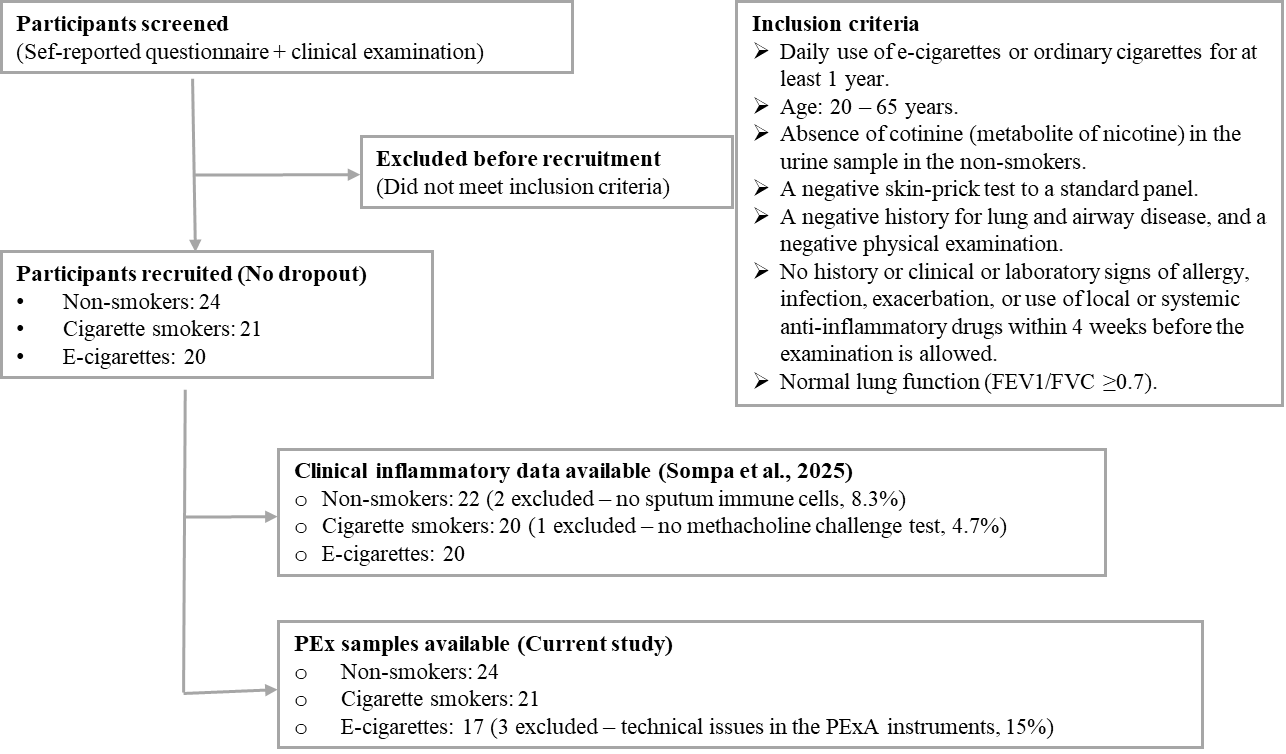


**Figure S1:** The flow chart shows the recruitment, exclusion, and inclusion process for participants. All volunteers were recruited after completing self-reported questionnaires and clinical exams, with only eligible individuals included. Hence, there were no dropouts after recruitment. A total of 24 non-smokers, 21 cigarette smokers, and 20 e-cigarette users participated. Variations in participant numbers across analyses are due to differences in available clinical data or biological samples. In the earlier study by Sompa et al. (2025), exclusions were made because some could not perform the methacholine challenge test (n=1 smoker) or lacked immune cells in induced sputum (n=2 non-smokers). For the current study, three e-cigarette users were excluded due to missing PEx samples caused by technical issues with the device. Percentages show exclusions relative to each exposure group's total recruited.
